# Factors associated with the appropriate use of ultra-broad spectrum antibiotics, meropenem, for suspected healthcare-associated pneumonia

**DOI:** 10.1097/MD.0000000000027488

**Published:** 2021-10-08

**Authors:** Nathan Kirsch, Jane Ha, Hee-Taik Kang, Tina Frisch, Ji Won Yoo, Craig Grossman, Neema Oroomchi, Hidenobu Shigemitsu, Chad L. Cross, Mutsumi John Kioka

**Affiliations:** a Division of Pulmonary and Critical Care Medicine, Department of Internal Medicine, University of Nevada, Las Vegas, NV; b Department of Medicine, Korea University College of Medicine, Seoul, Republic of Korea; c Department of Family Medicine, Chungbuk National University College of Medicine, Cheongju, Republic of Korea; d Department of Family Medicine, Chungbuk National University Hospital, Cheongju, Republic of Korea; e Department of Pharmacy University Medical Center, Las Vegas, NV; f Division of Geriatrics, Department of Internal Medicine, University of Nevada, Las Vegas, NV; g Department of Intensive Care Medicine, Tokyo Medical Dental University, Tokyo, Japan; h Department of Environmental and Occupational Health, University of Nevada, Las Vegas, NV.

**Keywords:** antibiotic stewardship, antibiotics, carbapenem, drug resistance, pneumonia

## Abstract

Pneumonia is a common disease-causing hospitalization. When a healthcare-associated infection is suspected, antibiotics that provide coverage for multi-drug resistant (MDR) or extended-spectrum beta-lactamase (ESBL) bacteria are frequently prescribed. Limited data is available for guidance on using meropenem as a first-line empiric antimicrobial in hospitalized patients with risk factors for MDR/ESBL bacterial infections.

This was a single-center, retrospective study designed and conducted to identify factors associated with positive cultures for MDR/ESBL pathogens in hospitalized patients with suspected healthcare-associated pneumonia.

Of the 246 patients, 103 patients (41%) received meropenem. Among patients prescribed meropenem, MDR/ESBL pathogens were detected in only 20 patients (13%). Patients admitted from a skilled nursing facility/long-term acute care (SNF/LTAC) or with a history of a positive culture for MDR/ESBL pathogens were significantly associated with positive cultures of MDR/ESBL pathogens during the hospitalization (odds ratio [95% confidence intervals], 31.40 [5.20–189.6] in SNF/LTAC and 18.50 [2.98–115.1] in history of culture-positive MDR/ESBL pathogen). There was no significant difference in mortality between the 3 antibiotic groups.

Admission from a SNF/LTAC or having a history of cultures positive for MDR/ESBL pathogens were significantly associated with a positive culture for MDR/ESBL pathogens during the subsequent admission. We did not detect significant association between meropenem use as a first-line drug and morbidity and mortality for patients admitted to the hospital with suspected healthcare-associated pneumonia, and further prospective studies with larger sample size are needed to confirm our findings.

## 1 Introduction

Bacterial resistance to antibiotics is a severe global health concern.^[1]^ Every year at least 4 million people acquire an infection with resistant bacteria, and about 23,000 people die from the associated complications.^[2]^ More recently the prevalence of multi-drug resistant (MDR) or extended-spectrum beta-lactamase (ESBL) organisms and the subgroups of MDR/ESBL organisms such as carbapenem-resistant enterobacteriaceae are raising public health concerns as these bacteria are associated with higher rates of morbidity and mortality.^[3]^

Although comprehensive data on the appropriateness of inpatient antibiotic use is lacking in the United States,^[4]^ 1 study found that 37% of antibiotics prescribed in sample hospitals across the country were potentially unnecessary.^[5]^ Inappropriate antibiotic use is associated with poor clinical outcomes and an increase in public health burdens, such as adverse drug reactions, the emergence of resistant pathogens, and cost.^[6]^ In many cases, physicians prescribe broad-spectrum antibiotics for presumed infections with MDR strains of bacteria. However, ultra-broad spectrum antibiotics such as carbapenems (meropenem) may be prescribed in fear of providing inadequate initial coverage since data for the management of sepsis has shown inadequate initial coverage is linked with significant increases in mortality.^[7–9]^ This study was designed to explore factors associated with MDR/ESBL pathogens in patients admitted with the diagnosis of healthcare-associated pneumonia (HCAP).

## 2 Methods

### 2.1 Study design, setting, and population

This study was approved with a waiver of informed consent by the Institutional Review Board of University of Nevada Las Vegas, Nevada (IRB No 1198024-1) and University Medical Center of Southern Nevada, Las Vegas, Nevada (IRB No UMC-2017-115).

We retrospectively analyzed data from an urban-based tertiary care teaching hospital from April 1 to December 31, 2018. Enrolled patients were 18 years or older and admitted to the medical/surgical care unit and medical intensive care unit (ICU) with a primary diagnosis of HCAP. The medical/surgical care unit is staffed with internal medicine and family medicine attending physicians, medical residents, and hospitalists The ICU is staffed with internal medicine residents, pulmonary/critical care fellows and attending physicians. The clinical diagnosis was made by the emergency department physicians and primary care teams at admission and documented in the patients’ chart. Patients were categorized into 3 groups identified as meropenem, cefepime, or piperacillin/tazobactam reflecting the initial antibiotic received. All patients empirically received an intravenous vancomycin dose to cover a presumed methicillin-resistant *Staphylococcus Aureus* infection. Cultures of sputum, blood, and urine taken during hospital admission were evaluated and patients with a primary infectious diagnosis other than HCAP such as gastrointestinal, skin, and urinary tract infection were excluded.

All patients were assessed to determine if they met the criteria of HCAP defined as hospitalization for at least 48 hours in the last 90 days, residence of a nursing home or long-term acute care (LTAC), received intravenous antibiotics, chemotherapy or wound care within the past 30 days, or attended a hemodialysis clinic.^[10]^ Furthermore, as to not influence the clinical decision-making process, the admitting team was not aware that their diagnoses and antibiotic choices were being monitored.

### 2.2 Statistical analysis

Continuous variables were compared using the Kruskal–Wallis test, and categorical variables were analyzed using a cross-tabulation chi-square test. To determine the independent risk factors for developing MDR/ESBL organisms in the cultures, logistic regression was used to test the following factors: admitted from a skilled nursing facility (SNF) or LTAC, history of tracheostomy, history of hemiplegia/paraplegia, age, Charlson comorbidity index, history of cultures positive for MDR/ESBL pathogens, history of multiple hospital admissions in the last 6 months, no risk factors for healthcare-associated infections, sex (male), and systemic inflammatory response syndrome score (SIRS)^[11]^ as previously described by others.^[12,13]^ Mean SIRS scores on day 1 and day 3 of hospitalization were compared with nonparametric tests (the Wilcoxon Signed Rank Test) for related samples. Use of at least 1 dose of meropenem was assessed to compare with cefepime or piperacillin/tazobactam whether it was associated with changing patients’ clinical outcomes such as overall mortality rate, in-hospital mortality rate, readmission rate, median length of hospital day, median length of ICU days, and median length of ventilator days in the subgroup of intubated patients. In all these analyses, the following factors were assessed to see if they might confound the relationship: age, sex, body mass index, Charlson comorbidity index, risk of mortality based on Mortality Probability Model III at zero hours score,^[14]^ and SIRS score. Analyses were performed utilizing SPSS (version 25; IBM Corp., Armonk, NY) and R (version 3.6.1; R-project, Vienna, Austria).

## 3 Results

### 3.1 Cohort and encounter characteristics

**Figure 1:**
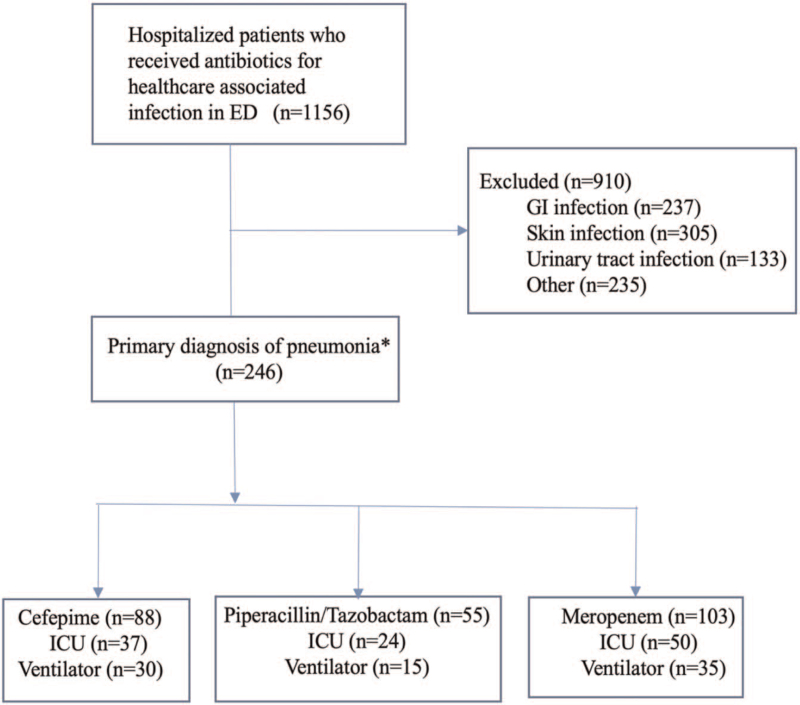
Cohort study flow diagram. ^∗^All study groups received intravenous vancomycin during the admission. ED = emergency department, GI = gastrointestinal, ICU = intensive care unit.

During the study period, 1156 patients were admitted to the hospital with a primary diagnosis of a healthcare acquired infection (Fig. 1). Of these, 910 patients were excluded due to a diagnosis of gastrointestinal, skin, or urinary tract infections, and 235 were excluded for other reasons. The remaining 246 patients had a diagnosis of HCAP who received meropenem (103 [41%]), cefepime (88 [36%]), or piperacillin/tazobactam (55 [22%]). Among the meropenem group, 50 were admitted to the ICU, and 35 underwent mechanical ventilation. Of the patients who received cefepime and piperacillin/tazobactam, 37 and 24, respectively, were admitted to the ICU, with 30 and 15 mechanically ventilated, respectively.

Characteristics of the study population are shown in Table 1. There were no significant differences between groups in age, sex, body mass index, MPM 0 score, history of tracheostomy, Charlson comorbidity index, hemiplegia/paraplegia, and SIRS score during the admission. The solid malignancy rate was significantly higher in the meropenem group (meropenem 26 [25%], piperacillin/tazobactam 9 [16%], cefepime 9 [10%]; *P* *=* .028). The admission rate from a SNF/LTAC was the highest in meropenem group (meropenem: 38 [37%], cefepime; 15 [17%], piperacillin/tazobactam: 11 [20%]; *P* = .004). The history of MDR/ESBL infection/colonization rate was highest in meropenem group but not statistically significant (meropenem: 10 [9.7%], cefepime: 2 [2.3%], piperacillin/tazobactam 3 [5.6%]; *P* = .101).

**Table 1:** Baseline demographic and clinical characteristics of patients by type of antibiotics.

	Cefepime	Piperacillin/tazobactam	Meropenem	
	(n = 88)	(n = 55)	(n = 103)	*P*-value
Age, years	60 (48–70)	55 (46–68)	61 (50–73)	.478
Sex, male	58 (65)	34 (62)	53 (51)	.121
BMI, kg/m^2^	25 (22–32)	25 (21–32)	25 (22–30)	.813
MPM 0, %	58 (22.3–80.0)	33 (16.9–58.6)	47 (20.6–67.7)	.461
SIRS, categories				.236
0	23 (26.1)	21 (38.9)	29 (28.2)	
1	40 (45.5)	19 (35.2)	46 (44.7)	
2	18 (20.5)	8 (14.8)	16 (15.5)	
3	2 (2.3)	2 (3.7)	0 (0)	
4	5 (5.7)	4 (7.4)	12 (11.7)	
No risk factor for healthcare-associated infection	52 (59)	19 (35)	31 (30)	<.001
Resident of SNIF/LTAC	15 (17)	11 (20)	38 (37)	.004
History of tracheostomy	8 (9.1)	1 (1.8)	10 (9.7)	.102
History of MDR/ESBL infection	2 (2.3)	3 (5.6)	10 (9.7)	.101
Charlson comorbidity index^∗^	4 (2–6)	4 (2–6)	5 (3–7)	.228
Arrhythmia	8 (9)	3 (5)	15 (15)	.187
Coronary artery disease	8 (9)	10 (18)	12 (12)	.245
Congestive heart failure	12 (14)	7 (13)	1 (1)	.443
Chronic kidney disease	16 (18)	9 (16)	20 (19)	.913
Chronic obstructive lung disease	20 (23)	9 (16)	26 (25)	.119
Dementia	10 (11)	3 (5)	16 (16)	.183
Hemiplegia/paraplegia	14 (15.9)	5 (9.3)	7 (6.8)	.123
Hypertension	43 (49)	29 (52)	38 (37)	.119
Leukemia/lymphoma	3 (3)	1 (2)	5 (5)	.629
Liver disease	4 (5)	5 (9)	0 (0)	.518
Solid malignancy	9 (10)	9 (16)	26 (25)	.028
Peptic ulcer disease	1 (1)	4 (7)	2 (2)	.072
Peripheral vascular disease	2 (2)	0 (0)	1 (1)	.468

Values are median (IQR) or number of patients (%). BMI = body mass index, ESBL = extended-spectrum beta-lactamase, IQR = interquartile range, LTAC = long term acute care, MDR = multi-drug resistant, MPM 0 = mortality probability model III at 0 hours, SIRS = systemic inflammatory response syndrome, SNF = skilled nursing facility.

∗ Harlson comorbidity index take into account following comorbidities: from arrhythmia to peripheral vascular disease.

Antibiotics prescription pattern and percentage of HCAP exclusion are shown in Table 5. More than 40% of each antibiotic was changed on day 1 (meropenem: 24 [43.6%], cefepime: 42 [47.4%], piperacillin/tazobactam: 24 [43.6%]; *P* = .404). HCAP exclusion percentage at the time of discharge from the hospital was the highest in piperacillin/tazobactam but not statistically significant (meropenem: 15 [14.6%], cefepime: 18 [20.5%], piperacillin/tazobactam: 17 [30.9%]). Comparison of clinical response on day 1 and day 3 done by SIRS score is shown in Table 6. There was no significant difference between day 1 and day 3 SIRS scores of each antibiotic group.

### 3.2 Microbial etiology

All culture samples were collected as a part of our hospital sepsis bundle on day 1. The overall results of microbial aetiology in the group of each antibiotic given in Table 2. The proportion of MDR/ESBL pathogens was significantly higher in the meropenem group (13%) compared to cefepime (5.6%) or piperacillin/tazobactam (3.6%) group (*P* = .012). In the meropenem group, 7.8% of organisms were carbapenem-resistant.

**Table 2:** Microbiological etiology.

Organisms	Cultures	Meropenem	Cefepime	Piperacillin/Tazobactam	*P*-value
Gram-positive cocci	53 (15)	20 (13)	17 (14)	16 (19)	.303
Gram-negative bacilli	57 (16)	27 (18)	17 (14)	13 (15)	.528
MDR/ESBL (+)^∗^	30 (8.3)	20 (13)	7 (5.6)	3 (3.6)	.012
CR (+)^†^	17 (4.7)	12 (7.8)	4 (2.9)	1 (1.2)	.038
CR (−)^†^	13 (3.6)	8 (5.2)	3 (3.2)	2 (2.4)	NA
Other^‡^	41 (11)	22 (14)	12 (9.7)	7 (8.3)	.295
No growth	212 (58)	86 (56)	78 (63)	48 (57)	.371
Total (n)^§^	363	153	124	84	

CR = carbapenem-resistant, MDR/ESBL = multi-drug resistant/extended-spectrum beta-lactamase, NA = not applicable.

∗ MDR/ESBL (+) numbers are included in the number of gram-negative bacilli.

† CR (+/−) numbers are included in the number of MDR/ESBL (+).

‡ Other: yeast, gram-positive bacilli, and anaerobic organisms.

§ Summation is greater than total because MDR/ESBL, CR (+/−) are included in Gram-negative bacilli.

### 3.3 Prescribing pattern of ultra-broad spectrum antibiotics (carbapenem) and predictors for developing MDR/ESBL organisms

As expected, a large portion of study patients (42%) with a diagnosis of HCAP received meropenem. Within the meropenem group, 31 (30%) of patients did not meet the criteria for HCAP as defined in the methodology, and regardless of the antibiotic group, more than one-third of the patients diagnosed with HCAP had only community acquired pneumonia (CAP) criteria (Table 1).

Significant predictors for developing MDR/ESBL organisms in cultures were admission from a SNF/LTAC, (odds ratio [OR], 23.29; 95% confidence interval [CI], 3.59–151.4; *P* = .001) and history of a positive culture for MDR/ESBL organisms (OR, 18.35; 95% CI, 2.88–117.1; *P* = .002) (Table 3). However, the rest of the variables were not significantly associated with developing MDR/ESBL pathogens. The Hosmer–Lemeshow test (HL) was not significant (HL = 5.513, *P* = .741), which indicates that the model was adequate. However, it should be noted that the OR and CI were wide, indicating a large standard error; this is often due to small sample sizes, and hence these results should be interpreted with that caveat in mind. Among the 3 groups, there was no significant difference in overall mortality (meropenem, 16%; cefepime, 18%; piperacillin/tazobactam, 9.3%; *P* = .349). No significant differences were identified in other outcomes or subgroup analysis of patients with/without risk factors for healthcare-associated infections in each antibiotic group (Table 4). These outcomes remained insignificant after controlling for potential confounders.

**Table 3:** Logistic regression analysis for developing MDR/ESBL pathogens in cultures.

Variables	OR (95% CIs)	*P*-value
Admit from SNF/LTAC	23.29 (3.59–151.4)	.001
History of tracheostomy	1.97 (0.51–7.62)	.328
History of positive MDR/ESBL pathogens in cultures	18.35 (2.88–117.1)	.002
Age	0.99 (0.96–1.03)	.692
Charlson comorbidity index score	1.03 (0.92–1.16)	.591
Hemiplegia/paraplegia	1.29 (0.31–5.39)	.730
History of multiple hospital admissions in the past 6 months	1.11 (0.76–1.33)	.591
No risk factor for healthcare-associated infection	0.69 (0.05–10.61)	.790
Sex, male	0.61 (0.18–1.99)	.409
SIRS Overall		.595
1^∗^	1.16 (0.24–5.58)	.850
2^∗^	2.72 (0.46–16.00)	.270
3^∗^	NA	NA
4^∗^	3.26 (0.46–23.29)	.238

ESBL = extended-spectrum beta-lactamase, LTAC = long-term acute care, MDR = multi-drug resistant, NA = not applicable, SIRS = systemic inflammatory response syndrome, SNIF = skilled nursing facility.

∗ ORs were based on comparison to SIRS 0. Patients with SIRS 3 were too sparse to calculate the CI. ^†^The Hosmer–Lemeshow test (HL = 5.513, *P* = .741).

**Table 4:** Clinical outcomes of patients.

	Overall series			
Clinical outcomes^∗^	Meropenem	Cefepime	Piperacillin/tazobactam	*P*-value
Overall mortality	16 (16%)	16 (18%)	5 (9.3%)	.349
Without risk factors	13 (18%)	8 (22%)	3 (8.6%)	
With risk factors	3 (9.7%)	8 (15%)	2 (11%)	
Overall total in-hospital mortality rate	14%	17%	7.4%	.263
Without risk factors	11 (15%)	7 (19%)	3 (8.6%)	
With risk factors	3 (9.7%)	8 (15%)	1 (5.3%)	
Overall readmission rate	36%	22%	28%	.091
Without risk factors	30 (42%)	11 (31%)	11 (31%)	
With risk factors	7 (23%)	8 (15%)	4 (21%)	
Overall median length of hospital days (IQR)	6 (3–11)	6.5 (3–10)	6.5 (3–13)	.939
Without risk factors	6.5 (3–11)	6.5 (2.25–14)	5 (3–14)	
With risk factors	5 (3–11)	6.5 (3.25–12.75)	7 (3–11)	
Overall median length of ICU days (IQR)	3 (1–5)	3 (2–9)	2 (1–9)	.267
Without risk factors	3 (1–6.5)	3.5 (1.75–10)	2 (1–9.25)	
With risk factors	2 (1–3)	3 (2–7.5)	2 (1–11)	
Overall median length of ventilator days (IQR)	6 (2–11)	6 (2–11)	6 (1–28)	.992
Without risk factors	6 (2.75–11.5)	5.5 (3–11.25)	4.5 (1.75–20.25)	
With risk factors	2 (2–13)	4 (2–13.5)	10 (1–29.5)	

ESBL = extended-spectrum beta-lactamase, ICU = intensive care unit, IQR = interquartile range, MDR = multi-drug resistant.

∗ A chi-square test of homogeneity and Kruskal–Wallis test were used for analyses of proportions and medians, respectively. Subgroup analysis for each clinical outcome was performed according to whether the patient had any risk factors of healthcare-associated infection include MDR/ESBL organisms.

## 4 Discussion

In the 2005 Infectious Disease Society of America guideline, the term HCAP was used to define pneumonia in a patient who is at high risk for MDR organisms because of a history of exposure to the healthcare system.^[10]^ In 2016, the guideline removed the term HCAP because the presence of MDR organisms was not found to be associated with risk factors for HCAP. One prospective study in a Spanish ICU found that 90% of patients diagnosed with HCAP could have been appropriately treated with therapy for CAP using antibiotics such as ceftriaxone and azithromycin.^[16]^ Therefore, the 2016 guidelines recommended empiric antibiotic coverage for MDR/ESBL organisms should be limited to hospital-acquired pneumonia which is pneumonia contracted by a patient in a hospital at least 48 to 72 hours after being admitted and ventilator-associated pneumonia, which is the sub-type of hospital-acquired pneumonia that occurs in people who are receiving mechanical ventilation.^[10]^ When a physician prescribes antibiotics to cover MDR gram-negative pathogens, the regimen should include piperacillin/tazobactam, cefepime, levofloxacin, imipenem, or meropenem. The guideline does not have a preferred antibiotic against pseudomonas because there has not been definitive evidence showing that one type of antipseudomonal agent is preferable over the others. However, meropenem is generally the drug of choice for MDR/ESBL infections, but there is also data suggesting that piperacillin/tazobactam or cefepime may be appropriate for the treatment of specific isolates.^[15]^ In 2019, the American Thoracic Society Infectious Disease Society of America guideline for the treatment of adults with CAP strongly recommended not using HCAP risk factors as an indication for extended antibiotic coverage in adults with CAP.^[17]^

In our study, regardless of the type of antibiotic initially prescribed, more than one third of patients did not have a history of exposure to the healthcare system in the past 90 days. Surprisingly, 31 (30%) of the meropenem group only met criteria for CAP (Table 1). This finding was supported by 15 (14.6%) of the meropenem group excluded from HCAP diagnosis at the time of discharge. Median respiratory culture return days were 3 to 4 days in each group. However, most initial antibiotics were discontinued or changed in the first 2 days (Table 5). There was no significant difference between SIRS scores on day 1 and day 3 of admission on all groups (Table 6). This antibiotics prescription pattern suggests that most initial antibiotics were changed or discontinued not from respiratory culture results or the patients’ clinical response after a couple of days of hospitalization.

**Table 5:** Antibiotics prescription pattern and percentage of HCAP exclusion.

	Piperacillin/tazobactam	Meropenem	Cefepime	
	(n = 55)	(n = 103)	(n = 88)	*P*-value
First antibiotics changed on day one (%)	24 (43.6)	52 (50.5)	42 (47.7)	.404
First antibiotics changed or discontinued day	2 (1–4)	1 (1–3)	2 (1–3)	.445
Total antibiotics day	5 (3–10)	6 (3–9)	6 (3–12.5)	.131
Respiratory culture returned day	4 (3–4.25)	4 (3–4.5)	3 (3–4)	.250
HCAP excluded (%)	17 (30.9)	15 (14.6)	18 (20.5)	.052

Values are median (IQR) or number of patients (%). HCAP = healthcare associated pneumonia, IQR = interquartile range.

**Table 6:** SIRS score day 1 and day 3.

	SIRS (day 1)	SIRS (day 3)	^∗^*P*-value
Piperacillin/tazobactam (n = 55)	1.09 (1.191)	0.87 (0.894)	.213
Meropenem (n = 103)	1.22 (1.204)	1.06 (1.054)	.438
Cefepime (n = 88)	1.16 (1.027)	1.20 (0.966)	.461

Data are presented in mean (SD). SD = standard deviation, SIRS = systemic inflammatory response syndrome.

∗ *P*-value were calculated by Wilcoxon Singed Rank Test for related samples.

Several factors might influence a provider's decision to prescribe meropenem in the absence of a clear indication. First, prescribing meropenem could be associated with a patients’ residence. Our study suggests that the residents of a SNF/LTAC is a risk factor for developing MDR/ESBL organisms in their cultures (OR, 23.29; 95% CI, 3.59–151.4; *P* = .001) (Table 3). Table 1 shows that 37% of patients in the meropenem group were admitted from a SNF/LTAC. Malcom et al^[12]^ suggested that risk factors for MDR/ESBL organisms were increasing age, a history of hospital admissions, residence in a long-term care faculty, and a high Charlson comorbidity index.^[13]^

From clinical experience, our emergency department and admitting physicians may be aware of this trend for positive cultures of MDR/ESBL organisms among patients admitted from a SNF/LTAC. Second, although not significant, patients in the meropenem group had more than twice the frequency of a documented history of MDR/ESBL infections than other groups (meropenem, 10 [9.7%]; piperacillin/tazobactam, 3 [5.6%]; cefepime, 2 [2.3%]; *P* = .101). These patient histories also explain the high prescribing rate of meropenem for this group. Third, the current guidelines strongly emphasize that the best empiric antimicrobial regimens should be based on local antimicrobial susceptibilities. Not surprisingly, the diagnosis of HCAP was strongly associated with the use of carbapenems because our local antibiogram showed a 77% sensitivity for cefepime and piperacillin/tazobactam against *Pseudomonas aeruginosa* (see Table, Supplemental Digital Content 1 which describes bacterial sensitivity patterns of University of Medical Center of Southern Nevada). This antibiogram likely impacted the frequency of meropenem chosen as the initial antibiotic even without a clear diagnosis of HCAP. Thus, the initial diagnosis of HCAP was the most common reason for choosing meropenem.

This chart review did not show clear reasoning for this diagnosis in patients without a clear indication. Frequently, no clear rationale was documented as to how the diagnosis of HCAP was made. Fourth, many previous studies suggest that improper initial antibiotic selection for sepsis increased mortality,^[18]^ but in our study, there was no difference in clinical outcomes (Table 4). According to culture results (Table 2), only 10% of the meropenem group grew MDR/ESBL organisms during the admission. In other words, 90% of the meropenem group had negative cultures or grew non-MDR/ESBL organisms. Regardless of the antibiotic group, more than one-fourth of patients had only a SIRS score of zero (Table 1). Hence, the many patients did not meet any criteria for sepsis and could be treated without antibiotics.^[16]^ According to Welker et al^[19]^ the core quality measure of sepsis management of time to first antibiotic dose in less than 4 hours of presentation to the emergency department might increase pressure put on physicians. Thus, due to time constraints, providers could over-diagnosis pneumonia or possibly HCAP even when patients do not have risk factors for healthcare-associated infections.

Williams et al^[20]^ suggested that high Acute Physiology and Chronic Health Evaluation (APACHE) scores have a positive relationship with the number of antibiotics prescribed in patients, however, in our study the severity of disease cannot explain the ultra-broad spectrum antibiotic coverage because the SIRS score and Charlson comorbidity index score were not significantly different among the groups. Positive predictors of MDR/ESBL organisms were only residency in a SNF/LTAC (OR, 23.29; 95% CI, 3.59–151.4; *P* = .001) and history of a MDR/ESBL positive culture (OR, 18.35; 95% CI, 2.88–117.1; *P* = .002), but multiple hospital admissions in the past 6 months (OR, 1.11; 95% CI, 0.76–1.33; *P* = .591), history of tracheostomy (OR 1.97; 95% CI, 0.51–7.62; *P* = .328), hemiplegia/paraplegia (OR: 1.29; 95% CI, 0.31–5.39; *P* = .730), Charlson comorbidity index (OR: 1.03; 95% CI, 0.92–1.16; *P* = .591), and age (OR: 0.99, 95% CI, 0.96–1.03, *P* = .692) were not (Table 3). The probable explanation of this difference between prior studies and our results is the size of our patient population and characters.^[12]^

In patients without apparent risk factors for MDR/ESBL organisms, the drug chosen from among meropenem, cefepime, and piperacillin/tazobactam was not likely to clinically impact our patients. It is well known that the use of inappropriate or unjustified antibiotics is associated with resistance, adverse effects, and the development of secondary infections including *Clostridium difficile*.

Several limitations exist in our study. This is a single-center study, so the results may represent local practices that are not generalizable. Although 13% of the meropenem group grew MDR/ESBL pathogens, it is unclear what percentage of patients would truly benefit from meropenem because it is common to have culture-negative sepsis^[21]^ (Table 2). This study is limited by its retrospective design and small sample size which has weakened the statistical power. Regardless, these are intriguing findings that serve as hypothesis-generating results for further investigation using a larger sample sizes or for replication at other hospitals. Future prospective studies with sufficient sample size are warranted to confirm our findings.

In conclusion, for patients with a diagnosis HCAP, a high rate of overdiagnosis HCAP and inappropriate ultra-broad spectrum antibiotic (meropenem) were used for initial empiric treatment. There was no benefit of using carbapenem (meropenem) as a first-line drug for patients without a definite risk factor for an MDR/ESBL infections. Therefore, empiric antibiotics with carbapenems might be considered for select patients who are admitted from a SNF/LTAC or have a history of an MDR/ESBL infection or if a patient is at risk based on local community patterns.

## Acknowledgments

None.

## Author contributions

**Conceptualization:** Mutsumi john kioka.

**Data curation:** Nathan Kirsch, Tina Frisch, Craig Grossman, Neema Oroomchi, Chad Cross, Mutsumi John Kioka.

**Formal analysis:** Chad Cross.

**Investigation:** Tina Frisch, Ji Won Yoo, Mutsumi John Kioka.

**Methodology:** Ji Won Yoo, Chad Cross, Mutsumi John Kioka.

**Project administration:** Mutsumi John Kioka.

**Software:** Chad Cross.

**Supervision:** Hee-Taik Kang, Ji Won Yoo.

**Validation:** Jane Ha, Hee-Taik Kang, Ji Won Yoo, Hidenobu Shigemitsu, Mutsumi John Kioka.

**Visualization:** Mutsumi John Kioka.

**Writing – original draft:** Mutsumi John Kioka.

**Writing – review & editing:** Jane Ha, Hee-Taik Kang, Hidenobu Shigemitsu, Mutsumi John Kioka.

## Supplementary Material

Supplemental Digital Content
