# Supplementary material for: Factors associated with the appropriate use of ultra-broad spectrum antibiotics, meropenem, for suspected healthcare-associated pneumonia
Source: Medicine (Baltimore). 2021 Oct 8;100(40):e27488. doi: 10.1097/MD.0000000000027488 (PMC8500636; doi:10.1097/MD.0000000000027488)
Supplement: Supplemental Digital Content [file medi-100-e27488-s001.pdf]

Supplemental Digital Content 1. Table that illustrates bacterial sensitivity patterns of University Medical Center of Southern Nevada, Las Vegas, NV from Jan 1, 2017 to June 30, 2017.

**Drug**	**Pregnancy Category**	**Acinetobacter spp.**	**E. cloacae**	**E. coli**	**Klebsiella pneumoniae**	**Pseudomonas aeruginosa**	**Proteus mirabilis**
Amikacin	D	73%	100%	99%	93%	97%	100%
Amp/Sulb	B	33%		31%	50%		
Cefazolin	B			62%	59%		92%
Cefepime	B	33%	92%	78%	75%	77%	100%
Ceftriaxone	B		60%	77%	75%		100%
Ciprofloxacin	C	27%	100%	53%	75%	65%	92%
Gentamicin	D	33%	96%	83%	92%	86%	96%
Meropenem	B	27%	92%	98%	93%	74%	
Minocycline	D	13%					
Pip/Tazo	B	0%	67%	73%	61%	77%	100%
Tetracycline	D				64%		
Tobramycin	D	33%	96%	80%	88%	88%	96%
Trimeth/Sulfa	C		92%	92%			83%

All adult units were included, and urinary isolates were excluded.

Amp/Sulb, Ampicillin/Sulbactam; Pip/Tazo, Piperacillin/Tazobactam; Trimeth/Sulfa, Trimethoprim/Sulfamethoxazole
